# Supplementary material for: Plastome data reveal multiple geographic origins of Quercus Group Ilex
Source: PeerJ. 2016 Apr 21;4:e1897. doi: 10.7717/peerj.1897 (PMC4846808; doi:10.7717/peerj.1897)

Supplement-fig. S3-1: Median-Joining network based on the amplified part of the rbcL gene

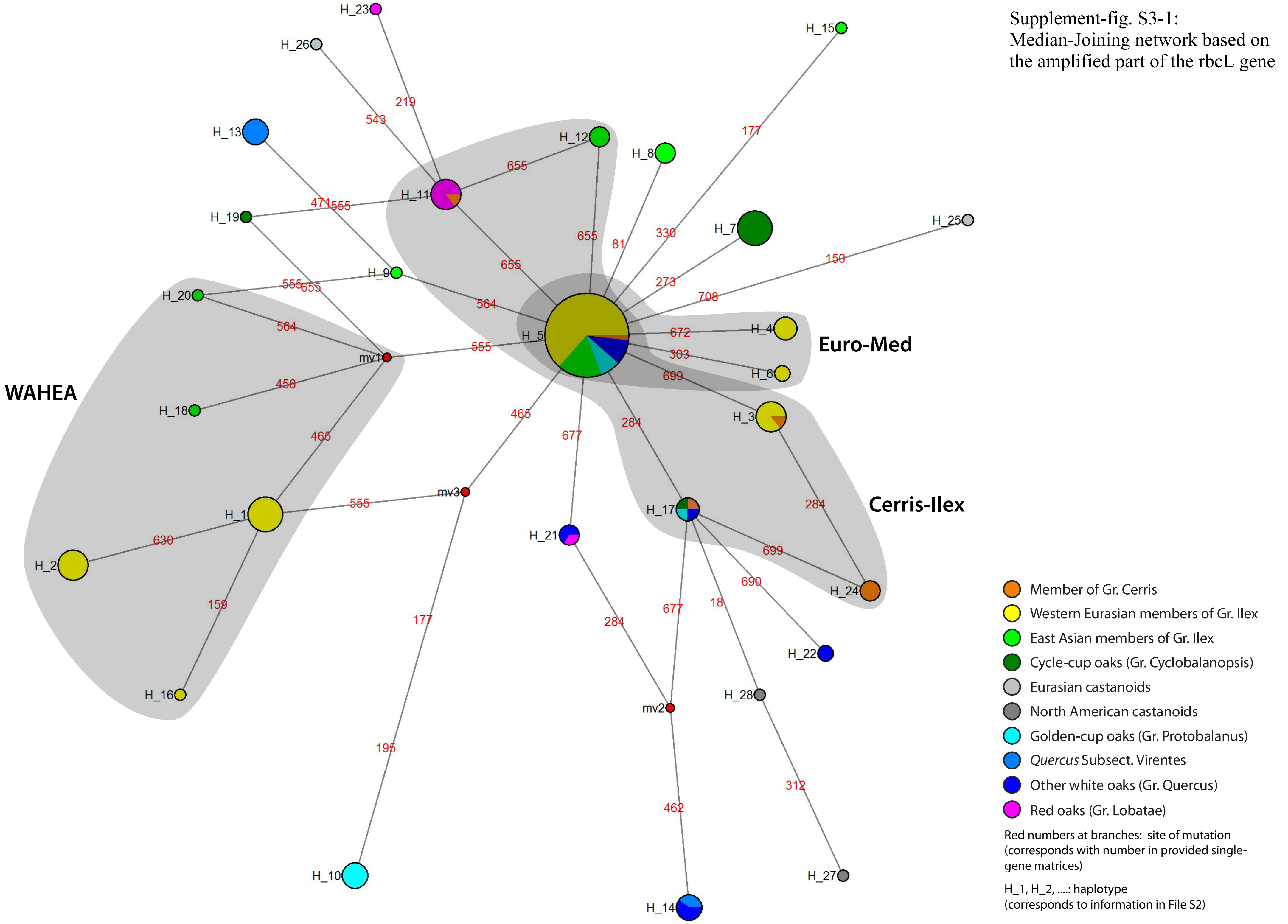

Supplement-fig. S3-2:
Median-Joining network based on
3' matK coding region

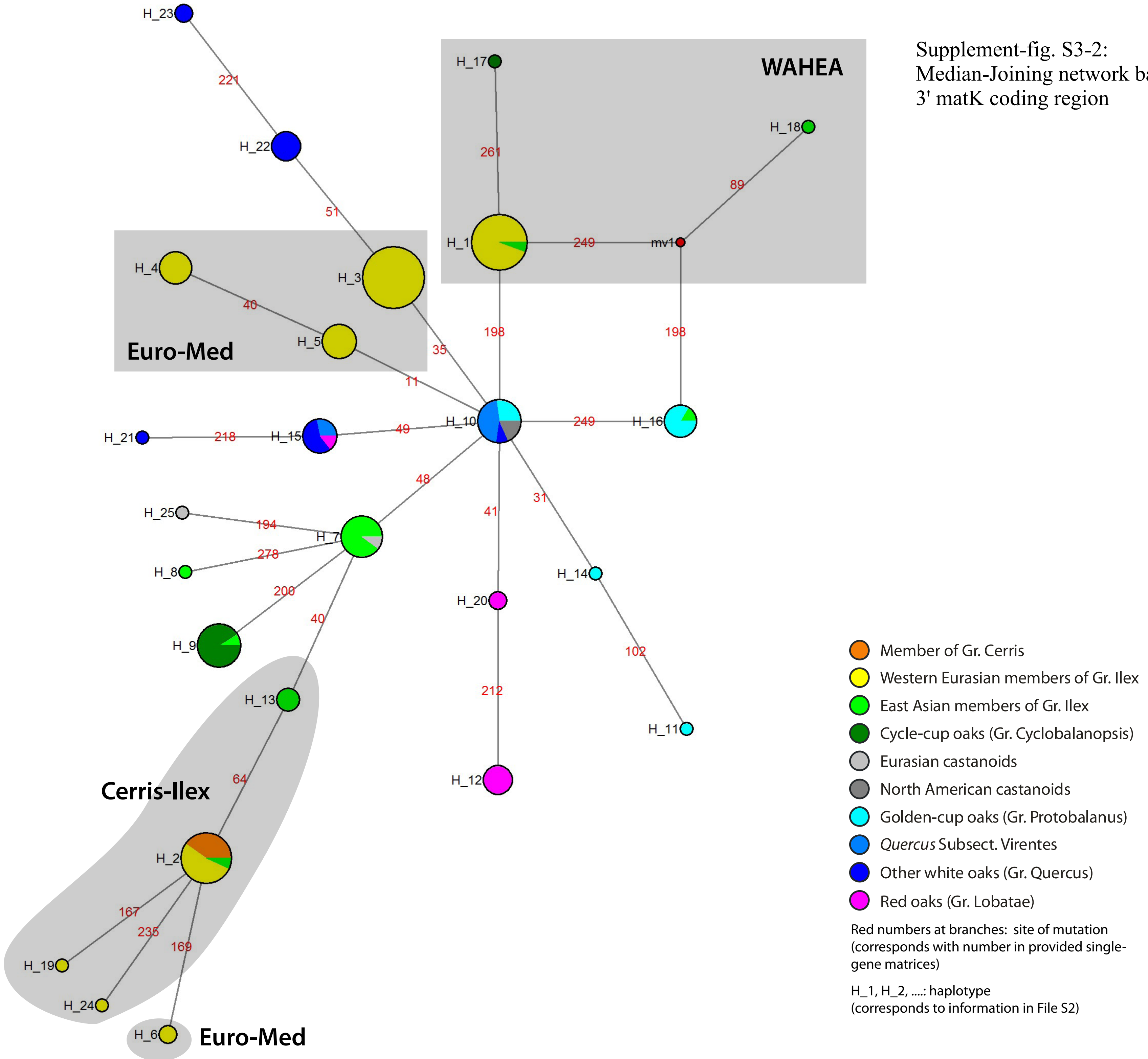

Supplement-fig. S3-3:
Median-Joining network based on
3' trnK intron

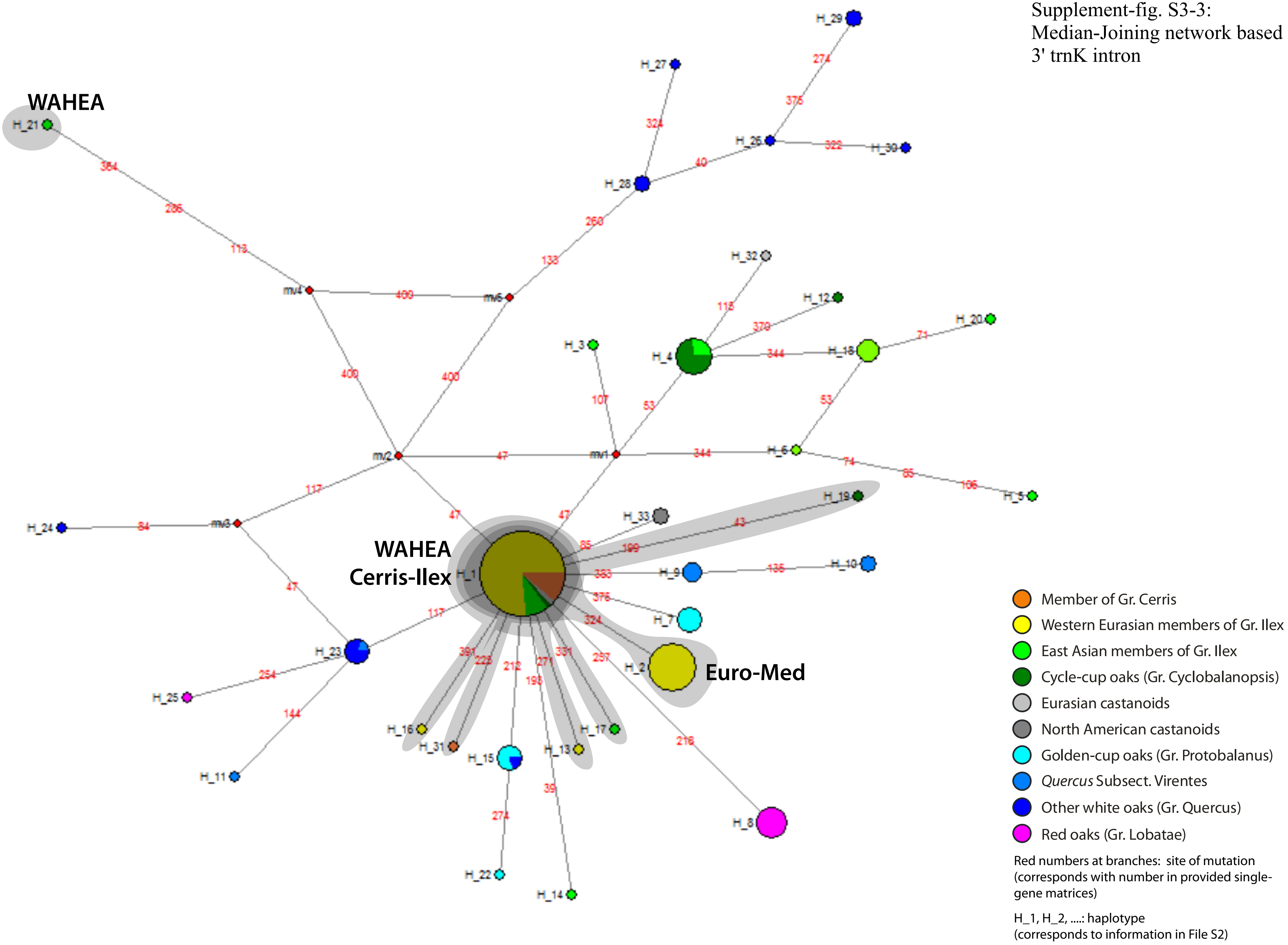

Supplement-fig. S3-4:
Median-Joining network based on
the combined matK/trnK strands

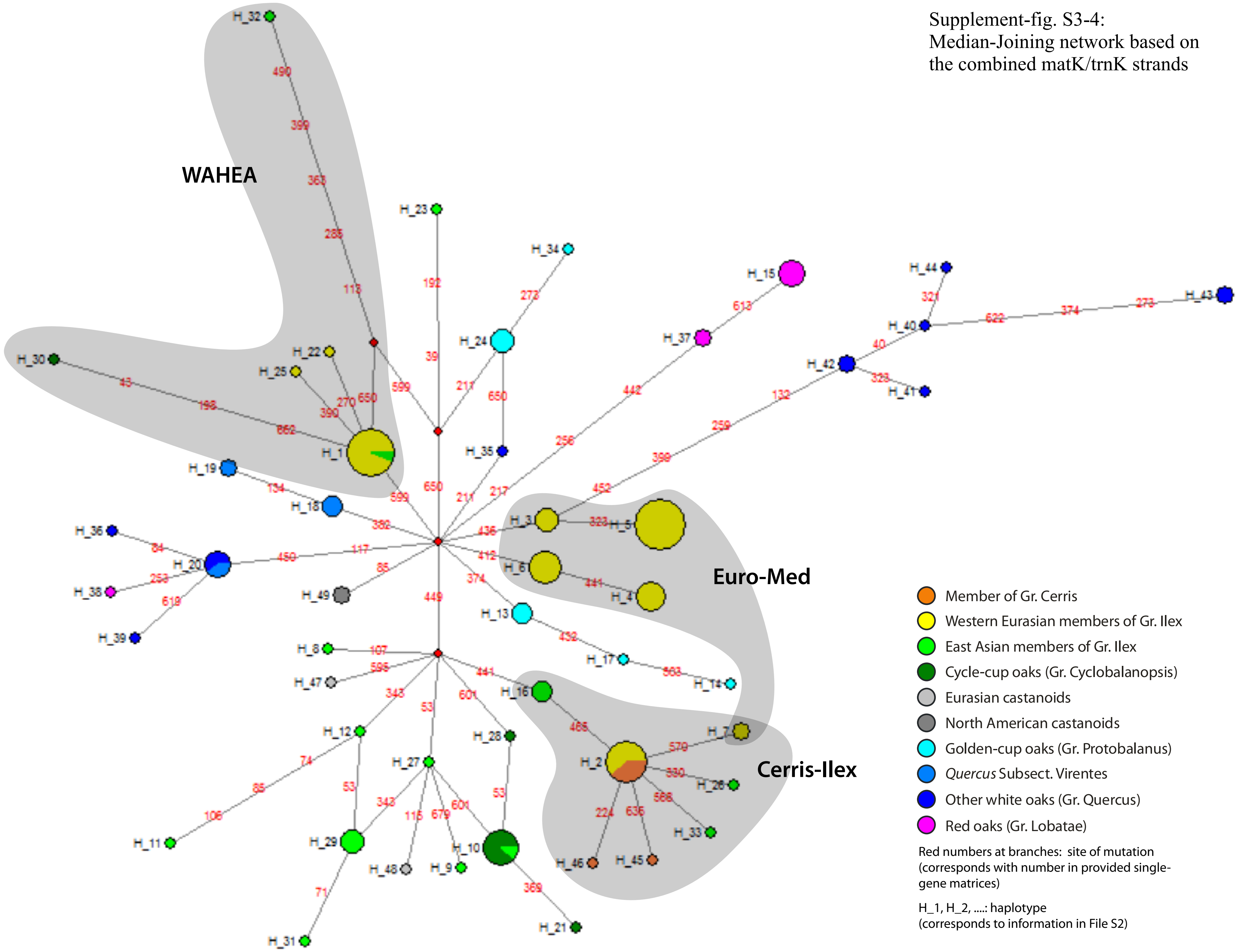

Supplement-fig. S3-5:
Median-Joining network based on
trnH-psbH spacer (gaps excluded; same
basic graph as shown in text-fig. 2)

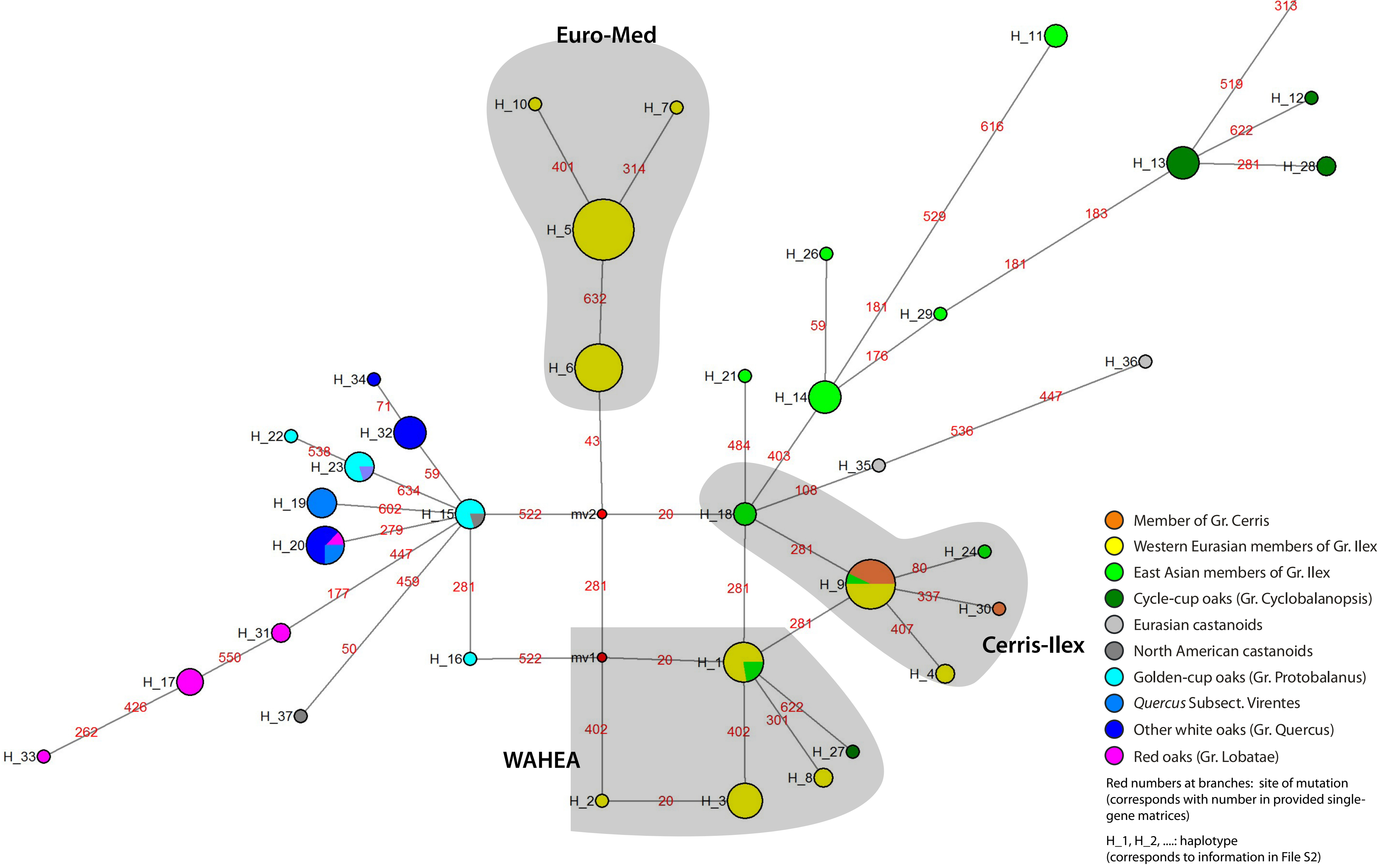

Supplement: File S3 — haplotype networks of the investigated dataset based on rbcL and trnK-matK markers. [file peerj-04-1897-s003.pdf]
